# *FPGS* rs1544105 polymorphism is associated with treatment outcome in pediatric B-cell precursor acute lymphoblastic leukemia

**DOI:** 10.1186/1475-2867-13-107

**Published:** 2013-10-29

**Authors:** Shu-Guang Liu, Chao Gao, Rui-Dong Zhang, Ying Jiao, Lei Cui, Wei-Jing Li, Zhen-Ping Chen, Min-Yuan Wu, Hu-Yong Zheng, Xiao-Xi Zhao, Zhi-Xia Yue, Zhi-Gang Li

**Affiliations:** 1 Hematology Oncology Center, Beijing Key Laboratory of Pediatric Hematology Oncology, National Key Discipline of Pediatrics, Beijing Children’s Hospital, Capital Medical University, 56 Nanlishi Road, 100045 Beijing, China

**Keywords:** Folypolyglutamate synthase (FPGS), Methotrexate (MTX), Polymorphism, Treatment outcome, Acute lymphoblastic leukemia (ALL)

## Abstract

**Background:**

Folypolyglutamate synthase (FPGS) catalyzes the polyglutamation of folates and antifolates, such as methotrexate (MTX), to produce highly active metabolites. *FPGS* tag SNP rs1544105C > T is located in the gene promoter. The aim of the present study was to investigate the impact of rs1544105 polymorphism on the treatment outcome in pediatric B-cell precursor acute lymphoblastic leukemia (BCP-ALL).

**Methods:**

This study enrolled 164 children with BCP-ALL. We genotyped the *FPGS* SNP rs1544105, and analyzed the associations between its genotypes and treatment outcome. We also examined *FPGS* mRNA levels by real-time PCR in 64 of the 164 children, and investigated the function of this polymorphism on gene expression.

**Results:**

We found significantly poor relapse-free survival (RFS) (*p* = 0.010) and poor event-free survival (EFS) (*p* = 0.046) in carriers of CC genotype. Multivariable Cox regression analyses adjusted for possible confounding variables showed that, relative to the CT + TT genotypes, the CC genotype was an independent prognostic factor for poor RFS (hazard ratio [HR], 4.992.; 95% CI, 1.550-16.078; *p* = 0.007). No association was found between any toxicity and rs1544105 polymorphism. Quantitative PCR results showed that individuals with the T allele had lower levels of *FPGS* transcripts.

**Conclusions:**

Our study indicates that *FPGS* rs1544105C > T polymorphism might influence *FPGS* expression and affect treatment outcome in BCP-ALL patients.

## Background

Cure rates for childhood acute lymphoblastic leukemia (ALL) have exceeded 80%. However, for the 15-20% of children with newly diagnosed ALL who will ultimately relapse, traditional risk assessment remains inadequate [1]. Multiple studies have suggested that the outcome in childhood ALL may be affected by how rapidly and effectively an individual patient metabolizes certain chemotherapeutic agents [2]. Thus, if the germline polymorphisms in drug-metabolism genes were better defined, tailored drug therapy based on these factors might further improve outcome.

Methotrexate (MTX) is a major component in all contemporary treatment protocols for childhood ALL. High-dose MTX (HDMTX) is associated with an improved outcome in ALL patients. Despite its clinical success, MTX may lead to several toxicities in a considerable number of patients [3,4]. Folypoly-γ-glutamate synthetase (FPGS) is responsible for the activation of MTX to MTX polyglutamates (MTXPGs). Loss of FPGS activity is an established mechanism of resistance to MTX *in vitro* and *in vivo*[5-7]. FPGS activity is significantly correlated with *FPGS* mRNA levels [8]. Overexpression of *FPGS* has been associated with increased sensitivity of several glioma cell lines to MTX [9]. In addition, altered *FPGS* expression was found associated with outcome of colorectal cancer patients [10]. In childhood ALL, a strong correlation exists between *FPGS* expression, intracellular MTXPGs accumulation and treatment outcome [8,11].

Up to now, there have been a few reports on the clinical importance of polymorphisms in *FPGS* gene [12-14]. However, its clinical role in childhood ALL remains to be defined. *FPGS* rs1544105 C > T is a tag SNP through HapMap database (release #27) in the promoter of *FPGS* gene. It is reported that *FPGS* rs1544105 C allele was associated with MTX poor response [13,14]. Panetta et al. found that rs1544105 polymorphism showed a significant relation to FPGS activity in childhood ALL [15]. So we hypothesized that rs1544105 might have important clinical roles in childhood ALL.

In the present study, we investigated the role of *FPGS* rs1544105 polymorphism in treatment outcome in children with newly diagnosed B-cell precursor ALL (BCP-ALL) and its influence on *FPGS* expression.

## Results

### Patient characteristics and *FPGS* genotyping

The main clinical characteristics of the 164 patients are shown in Table 1. *FPGS* rs1544105 genotype distribution is as follows: 14 CC (8.6%), 75 CT (45.7%), 75 TT (45.7%). Allele frequencies were in close agreement with values previously reported [13] and were in Hardy-Weinberg equilibrium (χ^2^ = 0.620, *p* = 0.431). In our study, rs1544105 genotypes had no association with the common clinical indices (Table 2).

**Table 1:** Baseline characteristics of patients with BCP-ALL (N = 164)

**Characteristics**	**N (%)**
Age (yr), median (range)	4
<1	0
1-9	136 (82.9)
≥10	28 (17.1)
Gender	
Male	104 (63.4)
Female	60 (36.6)
WBC (10^9^/L)	
<50	136 (82.9)
≥50	28 (17.1)
Treatment group	
SR	62 (37.8)
MR	81 (49.4)
HR	21 (12.8)
Fusion genes	
*TEL-AML1*	41 (25.0)
*E2A-PBX1*	16 (9.8)
*BCR-ABL*	8 (4.9)
*MLL-AF4*	1 (0.6)
CNS involvement	2 (1.2)
Event	
Relapse	16 (9.8)
Isolated BM	13 (7.9)
Isolated CNS	1 (0.6)
Isolated testis	2 (1.2)
Death*	4 (2.4)
Prednisone response	
Good	162 (98.8)
Poor	2 (1.2)

BCP-ALL, B-cell precursor ALL; WBC, white blood cell count; SR, standard-risk; MR, medium-risk; HR, high-risk; BM, bone marrow; CNS, central nervous system. *4 patients died of severe infections during induction.

**Table 2:** **The association between *FPGS *** **rs1544105 genotypes and patient characteristics (N = 164)**

**Characteristics**	***FPGS *** **SNP rs1544105**		*p*-value*
	**CC**	**CT + TT**	
	**genotype**	**genotype**	
Gender			1.000
Male	9 (62.3)	95 (63.3)	
Female	5 (35.7)	55 (36.7)	
Age (years)			0.336
<1	0	0	
1-9	13 (92.9)	123 (82.0)	
≥10	1 (7.1)	27 (18.0)	
Treatment group			0.797
SR	4 (28.6)	58 (38.7)	
MR	8 (57.1)	73 (48.7)	
HR	2 (1.3)	19 (12.6)	
WBC (10^9^/L)			0.710
<50	11 (78.6)	125 (83.3)	
≥50	3 (21.4)	25 (16.7)	
*TEL-AML1*			0.770
Positive	3 (21.4)	38 (25.3)	
Negative	11 (78.6)	112 (74.7)	
*E2A-PBX1*			1.000
Positive	1 (7.1)	15 (10.0)	
Negative	13 (92.9)	135 (90.0)	
*BCR-ABL*			0.141
Positive	2 (14.3)	6 (4.0)	
Negative	12 (85.7)	144 (96.0)	
*MLL-AF4*			1.000
Positive	0	1 (0.6)	
Negative	14 (100.0)	149 (99.4)	
Prednisone response			0.164
Good	1 (7.1)	1 (0.6)	
Poor	13 (92.9)	149 (99.4)	
CNS involvement			1.000
Absent	14 (100.0)	148 (98.7)	
Present	0	2 (1.3)	

*Data were calculated by χ^2^ test. WBC, white blood cell count; SR, standard-risk; MR, medium-risk; HR, high-risk; CNS, central nervous system.

### *FPGS* rs1544105 T allele might cause decreased mRNA levels

**Figure 1:**
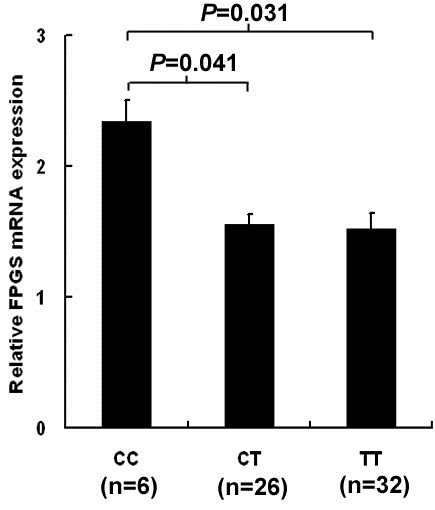
**The rs1544105 polymorphism influences mRNA levels of *FPGS*.** The expression level of *FPGS* was analyzed by quantitative RT-PCR and was normalized using *ABL* as reference gene. Data are shown as mean ± SEM.

As rs1544105 polymorphism is located in *FPGS* promoter, this polymorphism might impact the expression of *FPGS*. Therefore, we examined *FPGS* mRNA levels by real-time PCR in 64 children who had ≥70% leukemic cells in diagnostic bone marrow samples and sufficient cDNA for analysis, and whose *FPGS* rs1544105 genotypes were determined. As shown in Figure 1, we found that the *FPGS* rs1544105 T variant was associated with a relatively lower mRNA level. Average expression of patients harboring the CC genotype was approximately 1.5 times higher than CT (*p* = 0.041) and TT (*p* = 0.031) carriers. No significant differences were detected between CT and TT groups. In addition, *FPGS* mRNA expression had no association with gene fusions (Additional file 1: Table S1).

### *FPGS* rs1544105 polymorphism and treatment outcome

**Figure 2:**
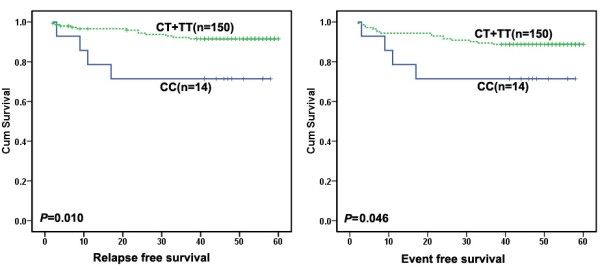
**The prognostic significance of *FPGS *rs1544105 in 164 BCP-ALL patients treated according to CCLG-ALL 2008 protocol.** The solid line was derived from the patients carrying CC genotype, dotted line from patients carrying CT and TT genotypes. There were different RFS (left, *p* = 0.010, by log-rank test) and EFS (right, *p* = 0.046, by log-rank test) between the two groups.

Given that rs1544105 polymorphism might influence *FPGS* mRNA levels, and altered *FPGS* expression was found associated with outcome of colorectal cancer patients [9], we analyzed the correlation between *FPGS* rs1544105 genotypes and outcomes in 164 patients. Figure 2 showed the effects of rs1544105 polymorphism on treatment outcome. There was a significantly higher relapse rate in CC group compared with CT + TT carriers (CC: 28.6%, CT + TT: 8.0%; χ^2^: *p* = 0.034). A poor RFS was found in the CC group (log rank: *p* = 0.010), as well as a poor EFS (log rank: *p* = 0.046). Multivariate survival analysis showed that the rs1544105 CC genotype was an independent prognosis factor for poor RFS (HR, 4.992; 95% CI, 1.550-16.078; *p* = 0.007) compared with the CT + TT genotypes (Table 3).

**Table 3:** Multivariate Cox-regression analysis for RFS and EFS in patients with BCP-ALL (N = 164)

**Variable**	**Multivariable analysis for RFS**			**Multivariable analysis for EFS**		
	**HR**	**95% CI**	***p*** *	**HR**	**95% CI**	***p*** *
Age						
<10	1	Reference	-	1	Reference	-
≥10	0.239	0.023-2.518	0.847	0.300	0.036-2.466	0.244
WBC (10^9^/L)						
<50	1	Reference	-	1	Reference	-
≥50	4.677	1.715-12.760	0.003	3.745	1.062-13.204	0.072
*BCR-ABL*						
Negative	1	Reference	-	1	Reference	-
Positive	7.190	2.022-25.567	0.118	2.701	0.497-14.681	0.250
*TEL-AML1*						
Negative	1	Reference	-	1	Reference	-
Positive	0.508	0.057-4.550	0.104	0.316	0.038-2.629	0.059
*E2A-PBX1*						
Negative						
Positive	1	Reference	-	1	Reference	-
	7.557	1.416-40.334	0.257	3.890	0.877-17.248	0.222
CNS involvement						
Absent	1	Reference	-	1	Reference	-
Present	25.259	5.124-124.504	0.001	11.375	1.487-86.991	0.019
rs1544105						
CT + TT	1	Reference	-	1	Reference	-
CC	4.992	1.550-16.078	0.007	2.723	0.766-9.674	0.059

*Data were calculated by Cox-regression analysis. BCP-ALL, B-cell precursor ALL; RFS, relapse free survival; EFS, event free survival; WBC, white blood cell count; CNS, central nervous system; HR, hazard ratio; 95% CI, 95% confidence interval.

### *FPGS* rs1544105 polymorphism and MTX-related toxicity

FPGS is responsible for the activation of MTX to MTXPGs and higher MTXPGs level is associated with increased toxicity [4]. Given that rs1544105 polymorphism might influence *FPGS* mRNA levels, so we next analyze the association between *FPGS* rs1544105 genotypes and MTX-related toxicities during consolidation therapy. As the HR group was treated with HD-MTX and other chemotherapeutic drugs, for example Dexamethasone, Vincristine, Cyclophosphamide, Cytarabine, L-asparaginase, which may influenced the assessment of MTX toxicity, only 139 patients enrolled in SR and MR treatment groups were evaluable for the toxicity analysis. As shown in Table 4, results showed that there was no correlation between any toxicity and rs1544105 genotypes, calculated by χ^2^ test.

**Table 4:** **The relationships between *FPGS *** **rs1544105 genotypes and MTX-related toxicity (SR and MR, N = 139)**

**Toxicity**	***FPGS *** **SNP rs1544105**		*p*-value*
	**CC (n,%)**	**CT + TT (n,%)**	
Oral mucositis			
With	1 (8.3)	11 (8.7)	0.969
Without	11 (91.7)	116 (91.3)	
Skin toxicity			
With	1 (8.3)	10 (7.9)	0.955
Without	11 (91.7)	117 (92.1)	
Hepatic toxicity			
With	0 (0)	17 (13.4)	0.176
Without	12 (100.0)	110 (86.6)	
Anemia			
With	6 (50.0)	52 (40.9)	0.543
Without	6 (50.0)	75 (59.1)	
Neutropenia			
With	6 (50.0)	77 (60.6)	0.473
Without	6 (50.0)	50 (39.4)	
Thrombocytopenia			
With	2(16.7)	25 (19.7)	0.801
Without	10 (83.3)	102 (80.3)	

*Data were calculated by χ^2^ test.

## Discussion

In the present study, we investigated whether *FPGS* rs1544105C > T polymorphism was associated with treatment outcome among childhood BCP-ALL patients. We found that CC genotype is significantly associated with poor RFS (*p* = 0.010) and EFS (*p* = 0.046) compared with the CT and TT genotypes. Furthermore, in multivariate Cox regression analysis, we found that the *FPGS* rs1544105 is an independent prognostic factor for RFS. Patients carrying the rs1544105 CC genotype displayed a higher risk of relapse than CT + TT genotypes (HR, 4.992; 95% CI, 1.550-16.078; *p* = 0.007), indicating that rs1544105 polymorphism is a potential predictor for survival in childhood BCP-ALL. Two previous studies reported that *FPGS* rs1544105 C allele was associated with MTX poor response in rheumatoid arthritis (RA) patients in north Indians [13,14], which were in line with our finding. To our knowledge, this is the first report that *FPGS* rs1544105 is associated with outcome in pediatric ALL, which extended our understanding of the role of *FPGS* in MTX response.

Previous observations suggested that FPGS downregulation leads to resistance to MTX and novel antifolates in some cancer cell lines [5,6,16,17]. As rs1544105 T allele carriers had lower FPGS mRNA level, it seems that they might be resistant to MTX therapy and had poor prognosis. However, our data suggested that T allele carriers had better RFS and EFS than CC genotype carriers, which appears to be in contrast with previous results. In fact, FPGS modulation affects both polyglutamylation of antifolates and folate cofactors [18-20]. Cho et.al reported that FPGS modulation on the chemosensitivity of breast cancer cells to MTX depends not only on MTX polyglutamylation but also on polyglutamylation of intracellular folate pools. So it is possible that *FPGS* rs1544105 T allele carriers had higher intracellular folate levels compared with CC genotype carriers, and consequently, increase in polyglutamylation of intracellular folate cofactors may diminish or even abolish the effects of decrease in polyglutamylation of antifolates. This might lead to enhanced chemosensitivity and better survival in T allele carriers. The differences of folate concentrations between T allele carriers and CC genotype carriers should be determined in the future.

Treatment-related toxicity can not only be life threatening, but is also one of the main reasons for interruption or discontinuation of chemotherapy, which may increase relapse risk [8,21]. Two previous studies reported that *FPGS* rs1544105 C allele was associated with MTX poor response in rheumatoid arthritis (RA) patients in north Indians [13,14]. In our study, no significant association was revealed between this polymorphism and any toxicity. The differences may result from our limited samples.

SNPs in gene regulatory region that alter the expression or function of proteins targeted by drugs can contribute significantly to variation in the responses of individuals. The rs1544105 is located in the promoter of *FPGS* gene, so we hypothesized that this polymorphism may affect treatment outcome by influencing *FPGS* mRNA expression. Among the164 genotyped patients, we analyzed the mRNA levels of 64 patients. We found that rs1544105 genotypes were correlated with mRNA levels of *FPGS*. The T allele carriers (CT + TT) had lower mRNA expression compared with CC genotype. Panetta et al. reported that FPGS activity of rs1544105 CC carriers was 2.6 times higher than CT and TT carriers in a St Jude ALL cohort [15]. As *FPGS* mRNA expression correlates with activity [8], their results were in agreement with ours. In order to understand the mechanism underlying the altering expression, we investigated the sequence for putative transcription factors binding sites using TRAP [22]. The studied SNP rs1544105 was located within a predicted binding site for cAMP response element-binding protein (CREB) transcription factors, and to modulate the binding affinity of CREB, with higher affinities predicted for the C-allele. CREB is a transcription factor that regulates gene expression principally through activation of the cyclic AMP (cAMP)-dependent cell signal transduction pathways by binding to the cAMP response element (CRE) region at the promoters. CREB is reported to be overexpressed in childhood ALL and be critical in leukemogenesis [23]. Further experiments are needed to reveal the detailed molecular mechanisms.

No statistical association was found between rs1544105 polymorphism and any clinical indices. It was reported that patients with *TEL-AML1* and *E2A-PBX1* fusion genes had significantly lower level of *FPGS* mRNA expression in childhood ALL. And TEL-AML1 and E2A-PBX1 can downregulate *FPGS* promoter activity and lead to lower mRNA expression and enzymatic activity [24,25]. As rs1544105 influences *FPGS* expression, we supposed that this polymorphism might correlate with *TEL-AML1*and *E2A-PBX1* fusions. However, we found no association between rs1544105 and *TEL-AML1* (*p* = 0.071) and *E2A-PBX1* (*p* = 0.601) fusions. Neither did we found any association between *FPGS* mRNA level and *TEL-AML1* (*p* = 0.070) and *E2A-PBX1* (*p* = 0.198) fusions. The difference may result from our limited samples. But our data suggested that *TEL-AML1*^*+*^ patients had lower FPGS mRNA expression than *TEL-AML1*^*-*^ ones (Additional file 1: Table S1), which was in agreement with previous results.

## Conclusions

*FPGS* rs1544105C > T polymorphism may influence the expression of *FPGS* and be associated with treatment outcome in childhood BCP-ALL. Larger prospective studies should be undertaken to confirm our recent results. In addition, the underlying mechanisms are still unclear and needs further study.

## Methods

### Study population

The criterion for patient inclusion in this study was newly diagnosed BCP-ALL patients who had at least 3-year follow-up time till March 2013 and sufficient DNA samples. Based on this criterion, 164 children enrolled during March 2008 to March 2010 and treated according to Chinese Children’s Leukemia Group (CCLG)-ALL 2008 protocol were collected in our study. All subjects were unrelated ethnic Chinese (aged 1.0-15.0 years, median 4.0 years). The common clinical characteristics of 164 patients were shown in Table 1. The median follow-up time was 48.0 months (range, 2.0 to 59.0) with 13 patients suffered bone marrow relapse, 1 suffered isolated central nervous system (CNS) relapse, 2 suffered isolated testis relapse, 4 died of severe infections during induction, 7 lost follow-up, and 137 cases were in continued complete remission (CCR).

Patients were stratified into standard-risk, medium-risk and high-risk treatment groups (SR, MR and HR) according to age, white blood cell count (WBC) in peripheral blood, immunophenotype, fusion gene, karyotype, CNS status, prednisone response, day 15 and day 33 BM remission status, minimal residue disease (MRD) on day 33 and at day 78, etc. (Additional file 2: Table S2). Sixty-two (37.8%) patients were assigned to SR group, 81 (49.4%) to MR group, and 21 (12.8%) to HR group (Table 1). Each group of patients received different treatment regimens. The details of treatment during the consolidation period are outlined in Additional file 3: Table S3. Doses of MTX differed by different treatment branch: the standard-risk (SR) group received 2 g/m^2^, while the medium- risk (MR) and high-risk (HR) groups received 5 g/m^2^. In addition, Non-HR (SR and MR) patients received oral 6-mercaptopurine (6-MP) and HR patients received other drugs concurrently. Leucovorin rescue was given at 42 h, 48 h and 54 h from the start of MTX infusion. Of the 164 patients in this study, 139 were enrolled into the analysis of association between *FPGS* SNP rs1544105 polymorphism and MTX-related toxicity during consolidation therapy. There were a total of 25 patients not analyzed for MTX-related toxicity, including 4 patients who died before consolidation therapy, and 21 HR patients who were treated with HD-MTX and other chemotherapeutic drugs, for example Dexamethasone, Vincristine, Cyclophosphamide, Cytarabine, L-asparaginase, which may influenced the assessment of MTX toxicity.

Bone marrow (BM) samples of patients were collected at diagnosis. Monouclear cell separation was carried out as previously described [4,26,27] and cells were immediately stored at −70°C until use. This study was approved by the BCH Institutional Ethics Committee and informed consent according to the Declaration of Helsinki was signed by the guardians of the patients.

### DNA extraction and SNP genotyping

Genomic DNA was extracted using a Genomic DNA Isolation Kit (U-gene, Anhui, China). Genotypes of the *FPGS* SNP rs1544105 were determined by PCR-RFLP methods [14]. *FPGS* rs1544105 primer sequences were forward: 5′-GTGCCTCCTTCACACACAG-3′and reverse: 5′-CCCAGAGTCCTTATTCTTAGCC-3′. PCR products were digested with HpyCH4IV (New England BilLabs, Inc, Beverly, MA) and separated on 10% polyacrylamide gel. A 10% random sample (n = 17) of the cases was verified by directly DNA sequencing of the PCR products.

### RNA extraction and FPGS expression analysis

The criterion for patient inclusion in expression analysis was ≥70% leukemic cells in diagnostic bone marrow samples and sufficient cDNA for analysis. Sixty-four patients among the 164 BCP-ALL genotyped in this study met this criterion and were included for a quantitative RNA expression assay. Total RNA from mononuclear cells was isolated using Trizol reagent (Invitrogen, Paisley, UK). The mRNAs were reverse-transcribed into cDNAs using random hexamers and moloney murine leukemia virus reverse transcriptase according to the manufacturer’s instructions (Promega, Madison, USA). The mRNA expression level of *FPGS* was measured by real-time quantitative reverse transcription-PCR using the ABI PRISM 7500 Sequence Detection System (Applied Biosystems, Singapore) based on the SYBR Green method. The *ABL* gene was used as the internal control, as suggested by the Europe Against Cancer program [28]. The primer sequences used for *FPGS* PCR were forward: 5′-CCCCGAGGTTCGAGTCTTG-3′ and reverse: 5′-GTCACTGTGAAGTTCTGTTGGTCTG-3′; and the primer sequences used for *ABL* were forward: 5′-TGGAGATAACACTCTAAGCATAACTAAAGGT-3′ and reverse: 5′-GATGTAGTTGCTTGGGACCCA-3′. The PCR reactions were performed in a total volume of 25 μl, including 12.5 μl of 2 × SYBR® Green PCR Master Mix (Applied Biosystems), 2 μl of cDNA, and 1 μl of primer mix (10 μM each). The PCR program was as follows: 95°C for 10 minutes, 40 cycles of 95°C for 15 seconds, 60°C for 1 minute, and a final step for generation of a dissociation curve to assess the specificity of the amplified products. Relative quantification analysis was performed using the comparative CT method (2^-△△CT^). Reactions were in triplicate in three independent experiments.

### Toxicity

Toxicities were graded according to the NCI Common Toxicity Criteria version 1.0. Hematological toxicity (anemia, thrombocytopenia, neutropenia) and non-hematological toxicity (skin toxicity, oral mucositis, and hepatic toxicity which means transaminase levels > 2 times as high as normal limit) were included. The highest grade of toxicity observed in each patient during the consolidation therapy period was recorded. No patients died from toxicity.

### Statistical analysis

The allele frequencies of polymorphisms were tested for Hardy-Weinberg equilibrium using the Chi-square (χ^2^) test. Toxicities were represented by the value 1or 0, indicating whether an adverse event did or not occur during the MTX course. χ^2^ test was used to determine the relationship between rs1544105 genotypes with toxicity, outcome and other clinicobiological characteristics. Relapse was defined as the reappearance of leukemic cells in BM (>5% blasts) and/or the reappearance of clinical evidence of the disease. Relapse-free survival (RFS) was defined as the time from CR to the date of relapse, censored at date of last time of contact or death in remission. The duration of event-free survival (EFS) was defined from the date of diagnosis to the date of relapse or death, or the last contact with patients in continuous hematologic CR. The probability of RFS and EFS were estimated using the Kaplan–Meier method and univariate associations between rs1544105 genotypes were compared by log-rank tests. Multivariate Cox regression models adjust for potential confounding variables were used to analyze the prognostic significance of rs1544105 genotypes. All statistical analyses were 2 tailed and performed with SPSS 16.0 for Windows. *P* value less than 0.05 were considered significant.

## Competing interests

The authors declare that they have no competing interests.

## Authors’ contributions

LSG and LZG designed the study. LSG performed the experiments, collected the data, and wrote the articles. GC, JY, CL, LWJ, ZXX, and YZX helped to collect the samples. ZRD, WMY, and ZHY recruited the patients. LSG, GC, JY and CZP participated in the statistical analysis. LZG reviewed the final manuscript and take primary responsibility for the article. All authors read and approved the final manuscript.

## Supplementary Material

Additional file 1: Table S1 The association between *FPGS* mRNA expression and gene fusions (N = 64). Click here for file

Additional file 2: Table S2 Stratification criteria of CCLG-2008 treatment protocol. Click here for file

Additional file 3: Table S3 Treatment elements of consolidation therapy in CCLG-ALL 2008. Click here for file
