# Supplementary material for: FPGS rs1544105 polymorphism is associated with treatment outcome in pediatric B-cell precursor acute lymphoblastic leukemia
Source: Cancer Cell Int. 2013 Oct 29;13:107. doi: 10.1186/1475-2867-13-107 (PMC3819686; doi:10.1186/1475-2867-13-107)
Supplement: Additional file 1: Table S1 — The association between FPGS mRNA expression and gene fusions (N = 64). [file 1475-2867-13-107-S1.doc]

Additional file 1: Table S1. The association between *FPGS* mRNA expression and gene fusions (N=64)

| Gene fusions | *FPGS* mRNA level† | *p-*value*** |
| --- | --- | --- |
| *TEL-AML1*  Positive  Negative | 1.246±0.212  1.719±0.118 | 0.070 |
| *E2A-PBX1*  Positive  Negative | 1.830±0.133  1.593±0.119 | 0.198 |
| *BCR-ABL*  Positive  Negative | 1.104±0.371  1.639±0.108 | 0.381 |

*Data were calculated by t-test. †Data were expressed as mean±SEM.
