# Supplementary material for: FPGS rs1544105 polymorphism is associated with treatment outcome in pediatric B-cell precursor acute lymphoblastic leukemia
Source: Cancer Cell Int. 2013 Oct 29;13:107. doi: 10.1186/1475-2867-13-107 (PMC3819686; doi:10.1186/1475-2867-13-107)
Supplement: Additional file 2: Table S2 — Stratification criteria of CCLG-2008 treatment protocol. [file 1475-2867-13-107-S2.doc]

Additional file 2: Table S2. Stratification criteria of CCLG-2008 treatment protocol

| Treatment group | Criteria |
| --- | --- |
| SR | 1≤age<10  WBC<50×10^9^/L  Good prednisone response  No T-cell and not mature B-cell  No t(9;22), t(1;19) or MLL rearrangements  Bone marrow morphology was M1/M2 at day15 and M1 at day 33  No CNSL  MRD<10^-4^ at day 33 |
| MR | Good prednisone response  No t(9;22) or MLL rearrangements  Bone marrow morphology at day15 was M1/M2 with MR protocol or M3 with SR protocol  MRD<10^-2^ at day 33 and <10^-3^ at day 78  Any one of:  Age<1  Age≥10  WBC≥50×10^9^/L  T-cell  CNSL with no other high risk factor |
| HR | Any one of:  Poor prednisone response  t(9;22) or MLL rearrangements  Bone marrow morphology was M2/M3 at day 33  MRD≥10-^2^ at day 33  MRD≥10^-3^ at day 78 |

WBC, white blood cell count; SR, standard-risk; MR, medium-risk; HR, high-risk; BM, bone marrow; CNSL, central nervous system leukemia; MRD, minimal residue disease.
