# Supplementary material for: FPGS rs1544105 polymorphism is associated with treatment outcome in pediatric B-cell precursor acute lymphoblastic leukemia
Source: Cancer Cell Int. 2013 Oct 29;13:107. doi: 10.1186/1475-2867-13-107 (PMC3819686; doi:10.1186/1475-2867-13-107)
Supplement: Additional file 3: Table S3 — Treatment elements of consolidation therapy in CCLG-ALL 2008. [file 1475-2867-13-107-S3.doc]

Additional file 3: Table S3. Treatment elements of consolidation therapy in CCLG-ALL 2008

| Treatment drug | Single or daily dose | Days of application |
| --- | --- | --- |
| SR  Methotrexate | 2 g/m^2^ per day, PI over 24 hours | 8, 22, 36, 50 |
| 6-mercaptopurine  Methotrexate | 25 mg/m^2^ per day, PO  12 mg/dose, IT | 1-56  8, 22, 36, 50 |
| MR  Methotrexate  6-mercaptopurine  Methotrexate | 5 g/m^2^ per day, PI over 24 hours  25 mg/m^2^ per day, PO  12 mg/dose, IT | 8, 22, 36, 50  1-56  8, 22, 36, 50 |
| HR (HR-1’/HR-2’/HR-3’) ×2  HR-1’  Dexamethasone  Vincristine  Methotrexate  Cyclophosphamide  Cytarabine  L-asparaginase  Methotrexate/cytarabine/prednisolone | 20 mg/m^2^ per day, PO  1.5 mg/m^2^ (max 2 mg), IV  5 g/m^2^ per day, PI over 24 hours  200 mg/m^2^ per dose, PI over 1 hours  2 g/m^2^ per dose, PI over 3 hours  25 000 U/m^2^ per day, PI over 2 hours  12/30/10 mg/dose, IT | 1-5  1, 6  1  2-4 (5 doses, 12-hour intervals)  5 (2 doses, 12 h interval)  6, 11  1 |
| HR-2’  Dexamethasone  Vincristine  Methotrexate  Ifosfamide  Daunorubicin  L-asparaginase  Methotrexate/cytarabine/prednisolone | 20 mg/m^2^ per day, PO  3 mg/m^2^ per dose (max 5 mg), IV  5 g/m^2^ per day, PI over 24 hours  800 mg/m^2^ per dose, PI over 1 hour  30 mg/m^2^ per dose PI over 24 hours  25 000 U/m^2^ per day, PI over 2 hours  12/30/10 mg/dose, IT | 1-5  1, 6  1  2-4 (5 doses, 12-hour intervals)  5  6, 11  1 |
| HR-3’  Dexamethasone  Cytarabine  Etoposide  L-asparaginase  Methotrexate/cytarabine/prednisolone | 20 mg/m^2^ per day, PO  2 g/m^2^ per dose, PI over 3 hours  100 mg/m^2^ per dose, PI over 1 hour  25 000 U/m^2^ per day, PI over 2 hours  12/30/10 mg/dose, IT | 1-5  1-2 (4 doses, 12-hour intervals)  3-5 (5 doses, 12-hour intervals)  6, 11  5 |

PO, orally; IV, intravenous push; PI, intravenous infusion; IT, intrathecally; SR, standard risk; MR, medium risk; HR, high risk.
